# Physical Inferiority and Crime

**Published:** 1897-02-27

**Authors:** 


### The Hospital

A Journal of

The Medical Sciences and Hospital Administration.

Vol. XXI.—No. 544.] [Feb. 27, 1897.

### Physical Inferiority and Crime.

It will not surprise any thoughtful medical man to be told, by so high an authority as the Rev. Mr. Morrison, editor of the "Criminology Series" and author of "Juvenile Offenders," that "physical inferiority," an inferior "physical basis of life," lies at the root of a larger proportion of juvenile crime than is due to any other single cause whatsoever. Drink is a cause of crime, even among children and youths; and it is often said to be the chief cause. But among the youthful offenders who are found in reformatories it is the fact that all but about 15 per cent. have spent the whole, or the greater portion, of their previous lives under defective and unwholesome economic conditions; under conditions, that is to say, which tend to stunted growth, poor physique, physical debility and instability, and, therefore, to mental feebleness and a readiness to yield to whatever impulses and temptations come in the way. The same is true of those who are found in industrial schools.

There is another thing to be said which will hardly surprise any who have kept themselves more or less "in touch" with all the literature and all the "tendencies" of the times. It is this: that so far as the facts are available, and they are extensively available in all civilised countries, crime by persons under adult age is almost everywhere on the increase; greatly on the increase in some countries, notably in America; less markedly in others, among which latter is England.

There are here two sets of facts for the thoughtful physiologist, as indeed for the thoughtful of all classes, to consider: first, the undoubted increase of juvenile crime; and, secondly, the equally demonstrable certainty that a physical cause lies at the root of most of this increase. There are two deductions to be drawn from these two sets of facts; deductions which if not necessarily contained in the logic are certainly contained in the imperative necessity of the situation. They are these: that if a defective physical basis of life be the chief cause of juvenile crime, the disease is curable; and further, that physiologists and physicians, the medical profession as a whole, is the department which must show the way to cure.

Now undoubtedly, so far as juvenile offenders themselves are concerned, prevention would be better than cure. That is to say, if all the children who live in surroundings which inevitably produce a poor physique and criminal mental endowments, could be removed from their surroundings and placed in adequately healthy conditions before the development of criminal tendencies—that would be the best of all methods of dealing with the case. It would be a kind of moral vaccination, which, if effectively carried out, would tend to extirpate juvenile crime altogether. But is such a course possible? At first sight it appears to be almost impossible, and in actual practice it might be found to be so, at any rate on an adequate scale. But this is the fact, that competent persons, schoolmasters and others who are in constant contact with the young, acquire a practical knowledge of them, which could not but be of the greatest possible national utility if it could but be acted upon. The criminal types among the young manifest themselves early; long before it is possible, or at any rate easy, for them to bring themselves within the controlling influence of the law. Would it not be feasible to offer to take these undeveloped criminals from their deadly environment, and, with the consent of their parents or guardians, to place them in institutions which should have no taint of criminal association about them, and, by healthy physical and mental environment, to "persuade," so to speak, the normal human virtues to grow in them, and so to crush and ki l by their growth all the undeveloped and abnormal tendencies to crime?

A hundred arguments can, of course, be advanced against this proposed moral vaccination in a moment. As, for instance, that it is hardly veiled socialism; that it is putting a premium upon the increase of the pauper and the criminal classes; that it is burdening the honest and well-doing in order that the ill-doing and the vicious may have no responsibility and no actual check upon their self-indulgence; and so on. It is impossible to deal with such arguments here and now; and, indeed, it is not necessary. Our one point for the moment is this, that when we are assured that a defective "physical basis of life" lies at the root of most of our juvenile crime; and when it is further insisted upon that the environment of much of our modern childhood is such that it must inevitably produce a physical basis of life which is defective; then, clearly, the appeal is not to religion, as in former times, nor to education, as in more recent years; but to physiology, to medicine, to sanitation. Put into a sentence or two, this is the condition of things: Juvenile crime is increasing the world over; the cause of the increase is a defective physical basis of life; remove the cause and the effect will cease. Who is to point the way to the removal of the cause? This is one of the most important problems of the day, and well worthy of consideration by the greatest minds.

